# Supplementary material for: Genetic mapping in the red mason bee implicates ANTSR as an ancient sex-determining locus in bees and ants
Source: PLoS Biol. 2025 Nov 3;23(11):e3003458. doi: 10.1371/journal.pbio.3003458 (PMC12594375; doi:10.1371/journal.pbio.3003458)
Supplement: Attachment — Submitted filename: Revision_response_to_reviewers.pdf [file pbio.3003458.s013.pdf]

Dear Editor,

Thank you for your valuable feedback and suggestions.
We have now updated the manuscript taking all of the comments into account.
To address the main suggestion, we've investigated evidence for increased nucleotide diversity around ANTSR in five more species: two halictid bees and three ants. This resulted in the addition of a new co-author. Please see below for a line-by-line response to each suggestion.

Editor's suggestions:

a) Please remove the Linnaean name of the study species from the Title, as it appears clearly in the Abstract: "Genetic mapping in the red mason bee implicates ANTSR as an ancient sex-determining locus in bees and ants"

The title of the manuscript has been updated to remove the Linnaean name.

b) Please address the reviewers' concerns. We discussed the more onerous requests from reviewer #2 with the Academic Editor; we think that for this Short Report format, the experimental (functional) work is beyond the scope of the study. However, it should be possible to include more genomes in the comparative analysis, and this should strengthen your findings - please let me know if you need additional time to do this. You should also make sure that you adjust any claims to be commensurate with the evidence presented.

Please see below for more in-depth responses to the reviewers' concerns.

c) Please address my Data Policy requests below; specifically, we need you to supply the numerical values underlying Figs 2ABCD, 3ABCD, 4, S1ABCD, S2AB, S3, either as a supplementary data file or as a permanent DOI'd deposition.
d) Please cite the location of the data clearly in all relevant main and supplementary Figure legends, e.g. "The data underlying this Figure can be found in S1 Data" or "The data underlying this Figure can be found in https://zenodo.org/records/XXXXXXXX
e) Please make any custom code available, either as a supplementary file or as part of your data deposition.

- All numerical values underlying the figures have been added to a Zenodo repository (DOI:10.5281/zenodo.17135872). Preview link to zenodo repository: https://zenodo.org/uploads/17135872?token=eyJhbGciOiJIUzUxMiIsImlhdCI6MTc1OTQwMDQ1NiwiZXhwIjoxNzYxOTU1MTk5fQ.eyJpZCI6IjQ1NmE2MjU5LWMxZGEtNDkyYS1hOGRkLTA1MjY5MGEzMjZjNSIsImRhdGEiOnt9LCJyYW5kb20iOiI0ZWQ1YmNiYmVhYzAxMjQ1MmM4ZWYxYmI1NWQ2NzVkNSJ9.8PTenpybcjM1kynd9KUdfwjouWy1VwH0fTYNGqLyhb0U1OqAboVrbDTXeHtk3qGUbw7KWpO9YWRSBb7TjrSp9A
-
- All figure legends have been updated to clearly indicate which supplementary data-table contains the numerical data underlying the figure.

- We've updated the Data-availability section on lines 437-447 to indicate where the supplementary data can be found.
- We've also included a tar.gz archive of the github repository with the supplementary data to provide an immutable snapshot of the analysis code.

Response to Reviewer 1:

We thank Reviewer 1 for their thoughtful and positive evaluation of our work. We are very grateful for their endorsement of the manuscript.

Response to Reviewer 2:

We thank Reviewer 2 for their thoughtful and positive evaluation of our work and helpful suggestions. We have addressed their concern as followed:

[...]This is an impressive piece of genomic and bioinformatic research but I think the conclusion goes beyond the data. I suggest two ways to strengthen this paper. First, functional genomic analysis to establish causality. This was done for the honeybee way back in 2003, with RNAi. An alternative is to do additional comparative genomic analyses. There are numerous genomes of bees and ants available, going beyond the few consulted in the present manuscript. Either or both would strengthen the ability to make the desired conclusion, especially for a top-tier journal like PLOS Biology.

In accordance with the suggestion of the Editor, we have opted for the secondary approach and added more species to the comparative genomics analysis to cover more of the Hymenoptera phylogeny. Ultimately, we identified five additional datasets suitable for our purpose: Two halictid bees, *Lasioglossum pauxillum* and *Lasioglossum albipes* and three ant species, two Formicinae, and one Myrmicinae: *Formica polyctena*, *Formica aquilonia* and *Solenopsis invicta*, respectively. In all investigated species, a local maximum of nucleotide diversity in the top 1% of 10 kbp bins across the genome is found in close vicinity of the ANTSR ortholog, which supports the action of balancing selection at this locus. In halictid bees ANTSR directly overlaps the maxima. In the Formicinae and Myrmicinae investigated, ANTSR is 45 kbp and 15 kbp away from the top-scoring bin, respectively. We believe this is likely because the sex determining locus is not located in ANTSR itself, but is a regulatory region that has undergone shifts in location over evolutionary time. This is discussed in lines 277-283 of the discussion section.

We have updated Figure 4 and its legend to include this new analysis. Similarly, we have updated the text of the manuscript in the results (lines 192-210), Discussion (lines 226-231), materials and methods (lines 400-435) and data availability statement. The raw sequencing data for *L. pauxillum* is uploaded to NCBI but unpublished as of yet. The bioproject referenced will be made public with the publication of this manuscript. The reviewer link for the data on NCBI is here: https://dataview.ncbi.nlm.nih.gov/object/PRJNA1330011?reviewer=okhsibakjqbulgt02cjj90heg8
All other additional species were derived from public datasets and referenced with their Bioproject number in the data availability statement.

Technical requests:

1) We ask that a manuscript source file is provided at Revision. Please upload your manuscript file as a .doc, .docx, .rtf or .tex.
2) Please provide separate figure files in .tif or .eps format.

We have now provided a source .docx file for the manuscript, as well as have converted our .svg sourcefiles into .eps format.

3) We have noticed that you have uploaded Supporting Information files, but you have not included a list of legends. Please add a full list of legends for your Supporting Information files after the references list.

We have now added a legend for all supplementary material at the end of the document. Labelling is split between supplementary **table** S1-9 and supplementary **data** S1-25 to indicate whether a resource is directly provided as a supplementary table with the manuscript or as part of a larger Zenodo data-deposition that holds numerical data underlying the results rather than descriptive supporting information.

i) Figure 1: Please confirm whether you drew the images / clip-art within the figure panels by hand. If you did not draw the images, please provide (a) a link to the source of the images or icons and their license / terms of use; or (b) written permission from the copyright holder to publish the images or icons under our CC BY 4.0 license. Alternatively, you may replace the images with open source alternatives. See these open source resources you may use to replace images / clip-art:
- https://commons.wikimedia.org
- https://openclipart.org/

We confirm that the images were drawn by hand with the exception of the image silhouettes from *Osmia bicornis*, *Apis mellifera* and *Linepitherma humile*. These images were obtained from PhyloPic (T. Michael Keesey, 2025) and were contributed by Thomas Hegna (2012; Public Domain Mark 1.0 Universal), Melissa Broussard (2023; Attribution 4.0 International), and Jitte Groothuis (2022; Attribution 4.0 International), respectively. The silhouettes were not modified. Images under PDM 1.0 are free of copyright restrictions, and images under CC BY 4.0 are directly compatible with your CC BY 4.0 license.
We've also added a sentence to indicate the specific creators of each silhouette in accordance to CC BY 4.0 to the figure caption.
